# Selective glomerular hypofiltration is associated with glucometabolic disturbances in the general population

**DOI:** 10.1111/joim.70148

**Published:** 2026-08-12

**Authors:** Christopher Nilsson, Agne Laucyte‐Cibulskiene, Amra Jujic, Marcus A. Ohlsson, Gregor Guron, Maria K. Svensson, Maria Weiner, P. Andreas Jonsson, Johan Ärnlöv, Gunnar Engström, Anders Grubb, Anders Larsson, Martin Magnusson, Anders Christensson

**Affiliations:** ^1^ Department of Clinical Sciences Lund University Malmö Sweden; ^2^ Department of Nephrology Skåne University Hospital Malmö Sweden; ^3^ Department of Molecular and Clinical Medicine/Nephrology Institute of Medicine The Sahlgrenska Academy at the University of Gothenburg Gothenburg Sweden; ^4^ Department of Medical Sciences, Renal Medicine Uppsala University Uppsala Sweden; ^5^ Uppsala Clinical Research Center Uppsala Sweden; ^6^ Department of Medicine, Health and Caring Sciences Linköping University Linköping Sweden; ^7^ Department of Clinical Nephrology Region Östergötland Linköping Sweden; ^8^ Department of Public Health and Clinical Medicine Umeå University Umeå Sweden; ^9^ Division of Family Medicine and Primary Care Department of Neurobiology, Care Sciences and Society Karolinska Institute Stockholm Sweden; ^10^ School of Health and Social Studies Dalarna University Falun Sweden; ^11^ Department of Clinical Chemistry Skåne University Hospital Lund University Lund Sweden; ^12^ Department of Medical Sciences Clinical Chemistry Uppsala University Uppsala Sweden; ^13^ Department of Cardiology Lund University Malmö Sweden; ^14^ Wallenberg Centre for Molecular Medicine Lund University Lund Sweden; ^15^ Hypertension in Africa Research Team (HART) North‐West University Potchefstroom South Africa

**Keywords:** creatinine, cystatin C, diabetes, diabetic kidney disease, glomerular filtration rate, insulin resistance

## Abstract

**Background:**

Discordance between cystatin C‐ and creatinine‐based estimated glomerular filtration rate (eGFR) has been linked to adverse outcomes. A low eGFR_cys_/eGFR_cr_‐ratio, conceptualized as selective glomerular hypofiltration syndrome (SGHS), has been hypothesized to reflect early metabolic kidney vulnerability, although non‐glomerular filtration rate determinants may also contribute. We investigated associations of dysglycemia and insulin resistance with the eGFR_cys_/eGFR_cr_‐ratio in a general population.

**Methods:**

In 28,078 participants aged 50–64 years from the Swedish CArdioPulmonary bioImage Study, we assessed glycemic status (normoglycemia, prediabetes, and diabetes), hemoglobin A1c (HbA1c), fasting glucose, and insulin resistance (homeostatic model assessment of insulin resistance [HOMA‐IR]). Outcomes were the continuous eGFR_cys_/eGFR_cr_‐ratio and SGHS defined as eGFR_cys_/eGFR_cr_‐ratio <0.7. Multivariable linear and logistic regression adjusted for demographic, metabolic, cardiovascular, and kidney covariates; sensitivity analyses used alternative eGFR equations.

**Results:**

Mean eGFR_cys_/eGFR_cr_‐ratio declined across glycemic categories (normoglycemia 0.95, prediabetes 0.90, diabetes 0.85; *p* < 0.001). SGHS prevalence increased stepwise (6.5%, 10.7%, and 19.5%, respectively). In fully adjusted models, higher HbA1c (per 10 mmol/mol) and fasting glucose (per 1 mmol/L) were inversely associated with the eGFR_cys_/eGFR_cr_‐ratio (*β* −0.015 and −0.008, respectively; both *p* < 0.001) and positively associated with SGHS (odds ratio [OR] 1.27 and 1.12; both *p* < 0.001). Higher HOMA‐IR was independently associated with SGHS, with the strongest associations observed in normoglycemia (OR 1.27, 95% confidence interval [CI] 1.12–1.45) and prediabetes (OR 1.41, 95% CI 1.12–1.76).

**Conclusions:**

Dysglycemia and insulin resistance were associated with a lower eGFR_cys_/eGFR_cr_‐ratio and higher SGHS prevalence. These findings may reflect kidney vulnerability, altered biomarker metabolism, or both.

## Graphical abstract

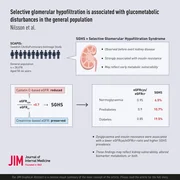

**Abbreviations**
BMI: body mass index
CAPA: Caucasian, Asian, pediatric, and adult (cystatin C equation)
CI: confidence interval
CKD‐EPI: Chronic Kidney Disease Epidemiology Collaboration
COPD: chronic obstructive pulmonary disease
CRP: C‐reactive protein
CVD: cardiovascular disease
DBP: diastolic blood pressure
DKD: diabetic kidney disease
EDTA: ethylenediaminetetraacetic acid
eGFR: estimated glomerular filtration rate
eGFR_cr_
: creatinine‐based estimated glomerular filtration rate
eGFR_cr‐cys_
: combined creatinine‐ and cystatin C‐based estimated glomerular filtration rate
eGFR_cys_
: cystatin C‐based estimated glomerular filtration rate
GFR: glomerular filtration rate
HbA1c: glycated hemoglobin A1c
HDL‐C: high‐density lipoprotein cholesterol
HOMA‐IR: homeostatic model assessment of insulin resistance
IQR: interquartile range
LDL‐C: low‐density lipoprotein cholesterol
LMrev: revised Lund–Malmö equation
OR: odds ratio
SBP: systolic blood pressure
SCAPIS: Swedish CArdioPulmonary bioImage Study
SD: standard deviation
SGHS: selective glomerular hypofiltration syndrome
SPS: shrunken pore syndrome

## Introduction

Metabolic disorders, such as dysglycemia and insulin resistance, are increasingly recognized as drivers of early organ dysfunction, often preceding clinically manifest disease [1]. Identifying subclinical markers of metabolic organ vulnerability is therefore central to risk stratification in both primary and secondary prevention. Diabetes is a major global public health challenge with steadily increasing prevalence. Diabetes is accompanied by multiple complications, with diabetic kidney disease (DKD) being the leading cause of kidney failure [2], requiring costly treatments such as dialysis or kidney transplantation. DKD substantially increases the risk of cardiovascular morbidity and mortality and accelerates progression to end‐stage kidney disease. Although cardiovascular disease (CVD) remains the leading cause of death in this population, the presence of DKD markedly amplifies this risk, making it one of the most critical prognostic factors for reduced survival [3]. Beyond overt diabetes, insulin resistance represents an early and prevalent metabolic disturbance that has been linked to endothelial dysfunction, microvascular injury, and altered kidney hemodynamics, even in individuals with normoglycemia [4]. However, how such early metabolic disturbances are reflected in routinely available markers of kidney function remains incompletely understood.

Kidney function is typically assessed using creatinine and/or cystatin C to estimate glomerular filtration rate (eGFR_cr_ and eGFR_cys_, respectively). Although eGFR is central to CKD diagnosis and staging, studies have highlighted discordance between these biomarkers [5]. Both creatinine and cystatin C are influenced by non‐glomerular filtration rate (GFR) determinants, although these differ between the two biomarkers [6]. Lower eGFR_cys_ relative to eGFR_cr_ is associated with adverse outcomes, including increased mortality, CVD, and progression to kidney failure, independent of overall kidney function [7]. This phenomenon was originally described by Grubb et al. in 2015 as *shrunken pore syndrome* (SPS), defined by a disproportionately low eGFR_cys_ compared to eGFR_cr_ (eGFR_cys_ <60% or 70% of eGFR_cr_) [5]. More recent work has broadened this concept to what has been termed *selective glomerular hypofiltration syndrome* (SGHS) [8, 9], proposed to reflect impaired filtration of mid‐sized molecules despite preserved filtration of small solutes, although the contribution of non‐GFR determinants remains uncertain [10, 11].

Whether eGFR biomarker discordance and SGHS are associated with early stages of metabolic dysfunction, including prediabetes and insulin resistance, remains unclear.

Therefore, in this study, we aimed to investigate the association of the eGFR_cys_/eGFR_cr_‐ratio and SGHS (defined as eGFR_cys_/eGFR_cr_ <0.7) with glucometabolic status (normoglycemia, prediabetes, or diabetes together with estimates for insulin resistance) in a study population selected from the general population. We hypothesized that metabolic dysregulation, particularly insulin resistance, is associated with eGFR biomarker discordance and SGHS.

## Methods

### Study population

We analyzed data from the population‐based Swedish CArdioPulmonary bioImage Study (SCAPIS), comprising individuals aged 50–64 years recruited at six centers (Gothenburg, Malmö, Stockholm, Umeå, Linköping, and Uppsala) during 2013–2018. The study aimed to recruit 30,000 randomly selected individuals aged 50–64 years from the Swedish population register, with approximately 5000 participants enrolled at each center. The participation rate was approximately 50% [12]. Of 30,154 participants, 29,246 had both creatinine and cystatin C available and were included.

### Study procedures and laboratory analyses

Study procedures have been described previously [12]. In the present study, data from physical examinations, questionnaires, and venous blood samples (after an overnight fast) were used. The following laboratory analyses were performed:

Hemoglobin A1c (*HbA1c*) was analyzed using high‐performance liquid chromatography at all sites, except for Malmö (turbidimetric method) and Uppsala (capillary electrophoresis). *Insulin* was measured in ethylenediaminetetraacetic acid (EDTA) plasma from frozen samples at a central laboratory using the Alinity I system (Abbott). *Lipids* (triglycerides, high‐density lipoprotein cholesterol, low‐density lipoprotein cholesterol [LDL‐C], and total cholesterol) were analyzed locally at each university hospital laboratory. *LDL‐C* was calculated using the Friedewald formula. *C‐reactive protein (CRP) was* analyzed using immunoturbidimetric methods (Cobas Roche) at all sites except Uppsala, where the Architect platform (Abbott) was used. *Creatinine* was analyzed from fresh samples using enzymatic colorimetric methods on Cobas analyzers (Cobas Roche) at all sites except Uppsala, where the Architect platform (Abbott) was used; where applicable, creatinine assays were isotope dilution mass spectrometry–traceable. *Cystatin C* was measured from frozen EDTA plasma using the Alinity C system (Abbott) with reagents (Gentian) and calibrated to the international reference preparation ERM‐DA 471/IFCC at a central lab. To account for calibration issues, a subset of 90 plasma samples was re‐analyzed at Uppsala University Hospital using Gentian reagents on a Mindray BS‐430 analyzer (Mindray). In parallel, pooled patient samples from the Caucasian, Asian, pediatric, and adult (CAPA) reference material [13] were included as controls. The comparison confirmed systematically lower concentrations in the original measurements. Based on the strong correlation between the two measurements (*r* ≈ 0.96–0.97), a correction formula was derived and applied in the present study: Corrected cystatin C (mg/L) = 0.187 + 0.955 × measured cystatin C.

All analyses were performed in accredited laboratories with standardized quality control.

### Glycemic status

Participants were categorized using World Health Organization thresholds based on fasting plasma glucose and/or HbA1c (single measurement). Categories included (1) *normoglycemic* (glucose <6.1 mmol/L and HbA1c <42 mmol/mol), (2) *prediabetes* (glucose between 6.1 and 6.9 mmol/L and/or HbA1c between 42 and 47 mmol/mol), and (3) *diabetes* (glucose ≥7.0 mmol/L and/or HbA1c ≥48 mmol/mol, or self‐reported diabetes).

### Assessment of insulin resistance

Based on fasting plasma glucose and fasting insulin, homeostasis model assessment of insulin resistance (HOMA‐IR) was calculated as HOMA‐IR = fasting insulin (µU/mL) × fasting glucose (mmol/L)/22.5 [14, 15].

### Estimation of glomerular filtration rate (eGFR)

GFR was estimated using the Chronic Kidney Disease Epidemiology Collaboration (CKD‐EPI) equations. The 2021 CKD‐EPI creatinine‐based equation [16] was applied for eGFR derived from creatinine (eGFR_cr_), whereas the 2012 CKD‐EPI cystatin C‐based equation [17] was used for eGFR derived from cystatin C (eGFR_cys_). The race‐free CKD‐EPI 2021 creatinine equation was selected because of its increasing clinical adoption. As performance may differ in European populations, sensitivity analyses were performed using revised Lund–Malmö equation (LMrev) [18] for creatinine and the “CAPA cohort” equation for eGFR_cys_ [13]. Models were adjusted for combined eGFR_cr‐cys_ (CKD‐EPI 2021 equation) to account for underlying kidney function. Because this variable incorporates both filtration markers, supplementary analyses without eGFR_cr‐cys_ adjustment were also performed and yielded similar results. Primary analyses were repeated using LMrev and CAPA equations.

### Statistical analyses

Approximately normally distributed continuous variables were summarized using means and standard deviations, whereas skewed variables were summarized using medians and interquartile ranges based on visual inspection of their distributions. Categorical variables were summarized by counts. Baseline tables were presented without *p*‐values to avoid misleading significance tests in large samples. Skewed variables were log‐transformed in regression analysis (creatinine, HOMA‐IR, smoking exposure, CRP, fasting insulin, waist‐to‐height ratio, and triglycerides). Complete data were available for 28,078 participants (96.0%), who constituted the analytic sample. Baseline characteristics were compared between included and excluded participants to assess potential selection bias (Table S15).

Associations with the continuous eGFR_cys_/eGFR_cr_‐ratio were examined using linear regression (*β*, 95% confidence interval [CI]), and associations with SGHS (eGFR_cys_/eGFR_cr_‐ratio <0.7) using logistic regression (odds ratios [ORs], 95% CI). All multivariable models included study site as a fixed effect and were adjusted for eGFR_cr‐cys_ (CKD‐EPI 2021) to account for underlying GFR differences. Age and HbA1c were scaled per 10 U, and smoking exposure was modeled as ln(pack‐years +1).

**Fig. 1:**
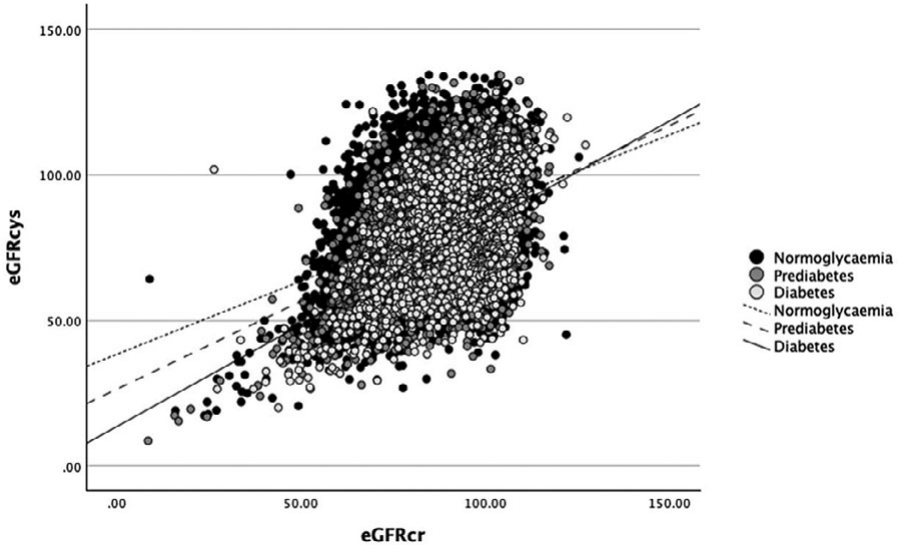
Relationship between creatinine‐based (eGFR_cr_) and cystatin C‐based (eGFR_cys_) estimated glomerular filtration rate by glycemic status. Each point represents one participant. Regression lines are shown separately for normoglycemia, prediabetes, and diabetes. For a given eGFR_cr_, eGFR_cys_ progressively declined across glycemic categories.

**Fig. 2:**
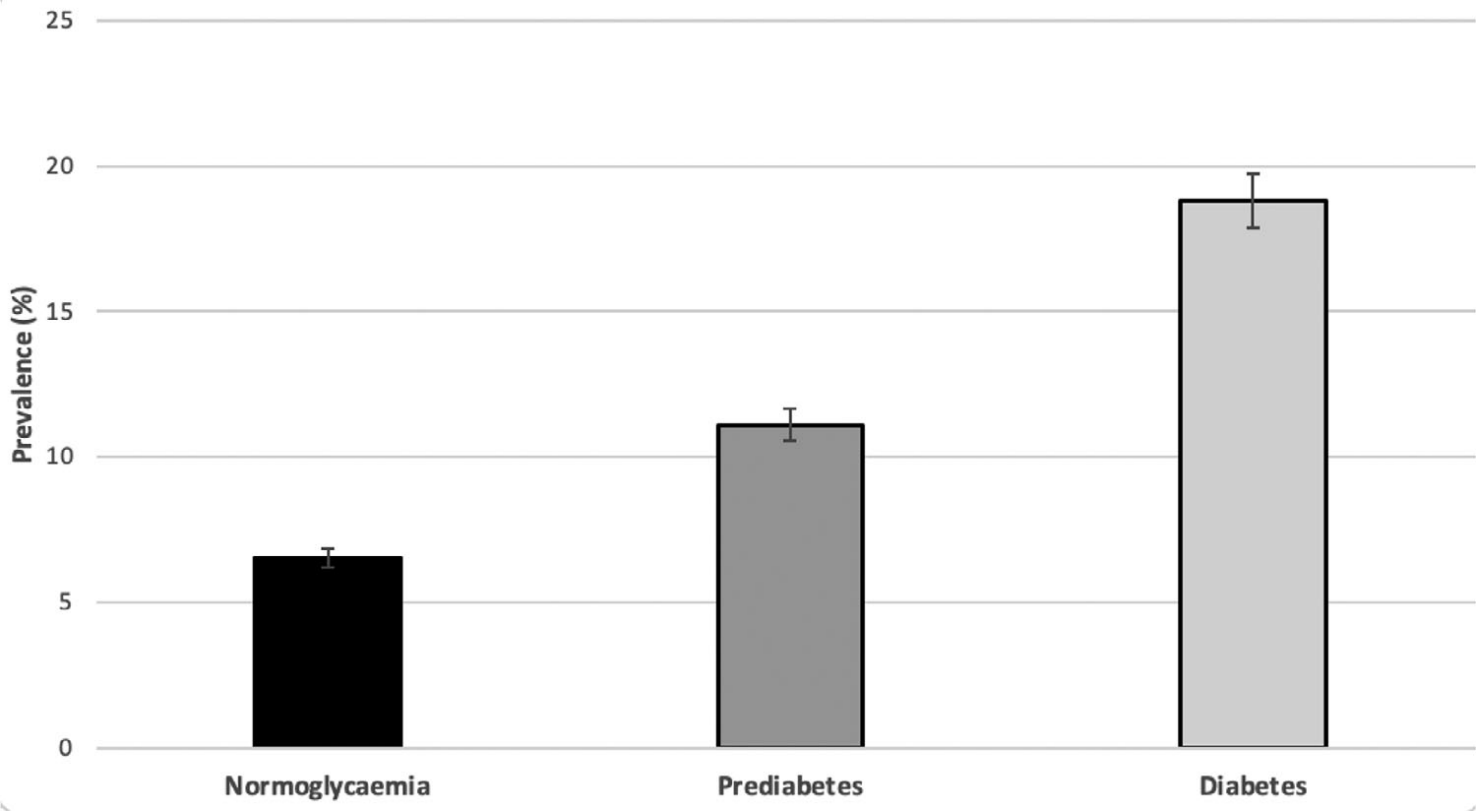
Prevalence of selective glomerular hypofiltration syndrome (SGHS), defined as an estimated glomerular filtration rate (eGFR)_cys_/eGFR_cr_‐ratio <0.7, across glycemic categories. eGFR was estimated using Chronic Kidney Disease Epidemiology Collaboration (CKD‐EPI) equations. Bars represent prevalence estimates, and error bars indicate 95% confidence intervals calculated using the Wilson method.

**Fig. 3:**
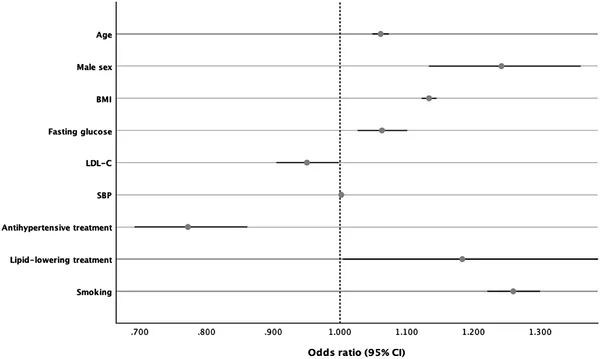
Forest plot depicting adjusted odds ratios (ORs) and corresponding 95% confidence intervals (CIs) for selected predictors of SGHS in the fully adjusted logistic regression model. Circles denote OR estimates, and horizontal bars represent 95% CIs. Values to the right of 1.0 indicate increased odds of SGHS, whereas values to the left indicate decreased odds. BMI, body mass index; LDL‐C, low‐density lipoprotein cholesterol; SBP, systolic blood pressure.

A prespecified hierarchical adjustment strategy was used, sequentially adding: (1) demographics and eGFR_cr‐cys_; (2) glycemic measures; (3) adiposity and smoking; (4) blood pressure and antihypertensive therapy; and (5) lipid profile and lipid‐lowering therapy. This framework was applied in both primary (CKD‐EPI) and sensitivity (CAPA/LMrev) analyses. To address concerns about potential overadjustment, parallel models without eGFR_cr‐cys_ were estimated, yielding similar effect estimates. Because HOMA‐IR is mathematically derived from fasting glucose, HOMA‐IR analyses were run in separate models where HOMA‐IR replaced glucose and HbA1c. Covariates were selected a priori based on well‐established associations with both glycemic status and markers of GFR. The stepwise modeling strategy allowed assessment of the stability of associations across increasing levels of covariate control, and no unexpected shifts or reversals were observed. Multiple sensitivity analyses using alternative adjustment sets further supported the robustness of the findings, suggesting that residual confounding is unlikely to fully explain the observed associations. Model diagnostics indicated no major violations of linearity or multicollinearity. A two‐sided *p* < 0.05 was considered statistically significant. Statistical analyses were performed in SPSS version 31 (IBM Corp.). Figures are prepared from the statistical output for graphical presentation. Figure 1 illustrates the relationship between eGFR_cys_ and eGFR_cr_, Fig. 2 displays SGHS prevalence with Wilson 95% CIs, and Fig. 3 presents adjusted ORs with 95% CIs.

### Ethics approval and consent to participate

The SCAPIS was approved by the regional ethics committee in Umeå (Dnr 2010‐228‐31 M) and complied with the Declaration of Helsinki. An additional ethical permit for this specific sub study was approved by the Swedish Ethical Review Authority (Dnr 2021‐01869). Written informed consent was obtained from all participants.

## Results

Baseline characteristics are presented in Tables 1 and 2. Of 29,246 participants with available creatinine and cystatin C measurements, 28,078 (96.0%) had complete data for all covariates included in the fully adjusted models and constituted the analytic sample. Participants excluded because of missing covariate data were overall broadly similar to those included in the analytic sample (Table S15). Age, HbA1c, fasting glucose, and the eGFR_cys_/eGFR_cr‐_ratio were nearly identical, whereas excluded participants were somewhat more often men and had slightly higher body mass index (BMI) and systolic blood pressure (SBP), suggesting limited selection bias related to missing covariate data. Prediabetes was present in 16.0% of participants, with the majority (3512 out of 4504) identified through impaired fasting glucose. Diabetes was observed in 7.1% of the population with 57.5% (1148 individuals) having a previously reported diagnosis. Compared with normoglycemia, participants with diabetes were older, more often men, and had higher BMI, blood pressure, and eGFR_cr_ but lower LDL‐C owing to more frequent lipid‐lowering therapy. eGFR_cr_ increased with worsening glycemic status, whereas eGFR_cys_ declined by glycemic status.

**Table 1:** Characteristics of the study population stratified by glycemic status.

Variable	All subjects	Normoglycemia	Prediabetes	Diabetes
**Subjects, % (*n*)**	100 (28,078)	76.8 (21,577)	16.0 (4504)	7.1 (1997)
***Basic characteristics***				
Age, years—mean (SD)	57.5 (4.3)	57.2 (4.3)	58.3 (4.3)	59.0 (4.1)
Male Sex—% (*n*)	48.7 (13,684)	46.4 (10,015)	53.7 (2418)	62.6 (1251)
BMI, kg/m^2^—median (Q1–Q3)	26.3 (23.9–29.4)	25.7 (23.4–28.4)	28.1 (25.7–31.1)	29.6 (26.9–33.1)
Waist circumference, cm—median (Q1–Q3)				
Men	99.0 (92.0–106.0)	97.0 (90.0–103.0)	102.0 (96.0–109.0)	106.0 (100.0–115.0)
Women	88.0 (80.0–97.0)	86.0 (79.0–94.0)	96.0 (88.0–104.0)	100.0 (92.0–110.0)
Waist‐to‐height ratio—median (Q1–Q3)				
Men	0.55 (0.51–0.59)	0.54 (0.50–0.58)	0.57 (0.54–0.61)	0.60 (0.56–0.65)
Women	0.53 (0.48–0.59)	0.52 (0.47–0.63)	0.58 (0.53–0.63)	0.61 (0.56–0.67)
Systolic blood pressure, mmHg—mean (SD)	125.8 (17.0)	124.2 (16.8)	130.4 (16.3)	132.4 (16.3)
Diastolic blood pressure, mmHg—mean (SD)	77.5 (10.5)	76.7 (10.5)	79.9 (10.1)	79.8 (9.7)
History of cardiovascular disease—% (*n*)	4.4 (1237)	3.3 (704)	6.6 (298)	11.8 (235)
History of COPD—% (*n*)	1.8 (513)	1.5 (319)	2.3 (106)	4.4 (88)
History of heart failure—% (*n*)	0.7 (190)	0.5 (98)	0.9 (44)	2.3 (48)
***Smoking status***				
Current smoking—% (*n*)	12.9 (3623)	12.1 (2576)	15.4 (694)	17.7 (353)
Ex‐smoker (regardless of cessation time)—% (*n*)	36.4 (10,220)	35.4 (7552)	39.2 (1827)	42.1 (841)
Pack years (ever smokers only)—median (Q1–Q3)	12.8 (5.5–23.3)	11.9 (5.0–21.8)	15.0 (6.8–27.0)	18.0 (10.0–30.8)
***Laboratory measurements***				
HbA1c, mmol/mol—mean (SD)	36.5 (6.3)	34.9 (3.0)	38.0 (3.8)	50.3 (14.0)
Fasting glucose, mmol/L—mean (SD)	5.6 (1.1)	5.3 (0.4)	6.0 (0.5)	8.0 (2.4)
Insulin, µU/mL—median (Q1–Q3)	5.28 (3.74–7.85)	4.72 (3.46–6.64)	7.37 (5.26–10.46)	10.44 (6.89–16.25)
HOMA‐IR—median (Q1–Q3)	1.29 (0.88–1.98)	1.11 (0.79–1.59)	1.93 (1.40–2.80)	3.50 (2.24–5.70)
Total cholesterol, mmol/L—mean (SD)	5.49 (1.05)	5.52 (1.02)	5.56 (1.06)	4.91 (1.19)
LDL‐C, mmol/L—mean (SD)	3.44 (0.96)	3.46 (0.94)	3.56 (0.96)	3.00 (1.07)
HDL‐C, mmol/L—mean (SD)	1.63 (0.50)	1.70 (0.50)	1.47 (0.43)	1.34 (0.44)
Triglycerides, mmol/L—mean (SD)	1.22 (0.77)	1.13 (0.71)	1.47 (0.78)	1.67 (1.00)
CRP, mg/L—median (Q1–Q3)	1.0 (0.6–2.2)	0.9 (0.6–1.9)	1.4 (0.7–2.9)	1.7 (0.8–3.6)
***Ongoing medication***				
Antihypertensive treatment—% (*n*)	19.1 (5349)	14.9 (3185)	26.9 (1212)	47.7 (952)
Lipid‐lowering treatment—% (*n*)	7.4 (2087)	4.8 (1034)	10.0 (451)	30.1 (602)
Glucose‐lowering treatment—% (*n*)	–	–	–	48.0 (959)
Insulin treatment—% (*n*)				15.9 (318)

Abbreviations: BMI, body mass index; CRP, C‐reactive protein; HDL‐C, high‐density lipoprotein cholesterol; IQR, interquartile range; LDL‐C, low‐density lipoprotein cholesterol; SD, standard deviation.

**Table 2:** Kidney‐specific characteristics of the study population stratified by glycemic status.

Variable	All subjects	Normoglycemia	Prediabetes	Diabetes
**Subjects, % (*n*)**	100 (28,078)	76.8 (21,577)	16.0 (4504)	7.1 (1997)
**Creatinine, µmol/L—median (Q1–Q3)**	77.0 (67.0–87.0)	76.0 (67.0–87.0)	79.0 (69.0–88.0)	76.0 (66.0–86.0)
**Cystatin C, mg/L—mean (SD)**	0.96 (0.17)	0.95 (0.16)	0.99 (0.20)	1.02 (0.21)
**eGFR_cr_ (CKD‐EPI), mL/min/1.73 m^2^—mean (SD)**	88.7 (12.2)	88.4 (12.1)	88.9 (12.3)	91.7 (13.0)
**eGFR_cys_ (CKD‐EPI), mL/min/1.73 m^2^—mean (SD)**	82.0 (15.8)	82.9 (15.4)	79.6 (15.2)	77.6 (17.5)
**eGFR_cr‐cys_ (CKD‐EPI), mL/min/1.73 m^2^—mean (SD)**	88.1 (13.3)	88.5 (12.9)	86.7 (13.9)	86.8 (15.3)
**eGFR_cys_/eGFR_cr_‐ratio (CKD‐EPI)—mean (SD)**	0.93 (0.18)	0.95 (0.18)	0.90 (0.17)	0.85 (0.18)
**eGFR <60 mL/min/1.73 m^2^—% (n)** ^a^	1.8% (512)	1.4% (296)	2.6% (123)	4.5% (93)
**SGHS (eGFR_cys_/eGFR_cr_‐ratio <0.7)—% (*n*) (CKD‐EPI)**	8.1% (2268)	6.5% (1396)	10.7% (483)	19.5% (389)
**eGFR_cys_ (CAPA) mL/min/1.73 m^2^—mean (SD)**	80.1 (15.9)	81.1 (15.7)	77.3 (15.6)	75.4 (16.7)
**eGFR_cr_ (LMrev) mL/min/1.73 m^2^—mean (SD)**	77.0 (9.7)	76.8 (9.5)	76.7 (9.8)	79.2 (11.4)
**eGFR_cys_/eGFR_cr_‐ratio (CAPA/LMrev)—mean (SD)**	1.05 (0.20)	1.06 (0.20)	1.01 (0.19)	0.95 (0.20)
**SGHS (eGFR_cys_/eGFR_cr_‐ratio <0.7), % (*n*) (CAPA/LMrev)**	1.8% (509)	1.3% (267)	2.6% (121)	5.8% (121)

Abbreviations: CAPA, Caucasian, Asian, pediatric, and adult cystatin C‐based estimating equation; CKD‐EPI, Chronic Kidney Disease Epidemiology Collaboration; eGFR, estimated glomerular filtration rate; LMrev, revised Lund–Malmö creatinine‐based estimating equation; SGHS, selective glomerular hypofiltration syndrome.

^a^Defined as an eGFR_cr‐cys_ **(CKD‐EPI) <60 mL/min/1.73 m^2^**.

### eGFR_cys_/eGFR_cr_‐ratio in relation to glycemic status

The eGFR_cys_/eGFR_cr_‐ratio declined stepwise across glycemic categories (0.95 in normoglycemia, 0.90 in prediabetes, and 0.85 in diabetes; *p* < 0.001, Table 2), a pattern replicated using the CAPA/LMrev equations. Figure 1 illustrates the strong correlation between eGFR_cys_ and eGFR_cr_ together with a progressive downward shift in the relationship between the two biomarkers across glycemic categories. In fully adjusted models, both HbA1c (*β* −0.015, 95% CI −0.018 to −0.012) and fasting glucose (*β* −0.008, 95% CI −0.010 to −0.006) were independently and inversely associated with the eGFR_cys_/eGFR_cr_‐ratio (Tables S1 and S2). These associations were robust to adjustment for eGFR_cr‐cys_ and to alternative eGFR equations (Tables S3 and S4). Among normoglycemic individuals, associations persisted but were attenuated (HbA1c *β* −0.009; glucose *β* −0.002; *p *< 0.001). Sex interaction analyses suggested stronger inverse associations in women for both glucose and HbA1c based on the direction of marginal estimates (HbA1c interaction *β* 0.007; glucose interaction *β* 0.003; *p* < 0.001).

### SGHS in relation to glycemic status

Overall, 8.1% of participants met the criteria for SGHS (eGFR_cys_/eGFR_cr_‐ratio <0.7). Prevalence increased stepwise with worsening glycemic status: 6.5% in normoglycemia, 10.7% in prediabetes, and 19.5% in diabetes (Table 2; Fig. 2). Using CAPA and LMrev equations yielded lower absolute prevalence but similar upward trends (1.3%, 2.6%, 5.8%; *p* < 0.001). In fully adjusted regression models (Model 5) as illustrated in Fig. 3, both HbA1c (per 10 mmol/mol) and fasting glucose (per 1 mmol/L) remained independently associated with SGHS (HbA1c OR 1.26, 95% CI 1.18–1.35; glucose OR 1.12, 95% CI 1.08–1.17), with full model results presented in Tables S1 and S2. Associations were weaker in men (interaction *p* < 0.001).

### Insulin resistance

Higher insulin resistance, assessed by HOMA‐IR, was associated with SGHS in normoglycemia (OR 1.27, 95% CI 1.12–1.45) and prediabetes (OR 1.41, 95% CI 1.12–1.76), whereas no significant association was observed in diabetes (OR 1.11, 95% CI 0.94–1.31). Excluding insulin‐treated participants within the diabetes group did not significantly alter these associations.

In fully adjusted linear regression, higher HOMA‐IR was also associated with a lower eGFR_cys_/eGFR_cr_‐ratio, with similar attenuation across glycemic categories. The strongest association was observed in normoglycemia (*β* −0.038, 95% CI −0.043 to −0.033), followed by prediabetes (*β* −0.039, 95% CI −0.049 to −0.030) and diabetes (*β* −0.012, 95% CI −0.021 to −0.004; Table 3). Results were consistent using alternative equations and when fasting insulin replaced HOMA‐IR (Tables S5 and S6).

**Table 3:** Homeostatic model assessment of insulin resistance (HOMA‐IR) and its association with selective glomerular hypofiltration syndrome (SGHS) and the estimated glomerular filtration rate (eGFR)_cys_/eGFR_cr_‐ratio (Chronic Kidney Disease Epidemiology Collaboration [CKD‐EPI] equations) stratified by glycemic status.

	SGHS (eGFR_cys_/eGFR_cr_‐ratio <0.7)		
	OR	95% CI	*p*‐value
Normoglycemia	1.273	1.119–1.450	<0.001
Prediabetes	1.405	1.120–1.762	0.003
Diabetes	1.110	0.939–1.311	0.221

	eGFR_cys_/eGFR_cr_‐ratio		
	*β*	95% CI	*p*‐value
Normoglycemia	−0.038	−0.043 to −0.033	<0.001
Prediabetes	−0.039	−0.049 to −0.030	<0.001
Diabetes	−0.012	−0.021 to −0.004	0.005

*Note*: Analysis was fully adjusted according to Model 5: Age, sex, study site, eGFR_cr‐cys_, BMI, smoking exposure, SBP, antihypertensive treatment, LDL‐C, and lipid‐lowering therapy. HOMA‐IR was log‐transformed before analysis.

Abbreviations: CI, confidence interval; OR, odds ratio.

### Additional sensitivity analysis

In sensitivity analyses where BMI was replaced by waist‐to‐height ratio, the associations with glycemic measures remained essentially unchanged. HbA1c was still inversely associated with the eGFR_cys_/eGFR_cr_‐ratio (*β* −0.012, 95% CI −0.012 to −0.012) and positively associated with SGHS (OR 1.17, 95% CI 1.15–1.18). Similar patterns were observed for fasting glucose (*β* −0.005, 95% CI −0.005 to −0.004; OR 1.05, 95% CI 1.05–1.06). To explore a potential role of inflammation, CRP was added to the fully adjusted models. Although CRP was independently associated with both outcomes, adjustment for CRP resulted in only minor attenuation of the observed associations (Tables S13 and S14).

To further evaluate the potential influence of non‐GFR determinants of cystatin C, sensitivity analyses, excluding participants with prevalent CVD and chronic obstructive pulmonary disease (COPD), yielded virtually identical results (Tables S9 and S10). When creatinine and cystatin C were modeled as separate outcomes (Tables S7 and S8), glycemic measures demonstrated divergent associations with the two markers. Higher HbA1c and fasting glucose were associated with lower creatinine concentrations, whereas both measures were positively associated with cystatin C. Several covariates showed similar bidirectional patterns: Male sex, BMI, smoking, SBP, and antihypertensive treatment were each associated with lower creatinine, but higher cystatin C. Results were unchanged in models without eGFR_cr‐cys_ adjustment (Tables S11 and S12).

## Discussion

In this large, population‐based study of nearly 30,000 middle‐aged individuals, we observed a progressive decline in the eGFR_cys_/eGFR_cr_‐ratio with worsening glycemic status, accompanied by a marked increase in the prevalence of SGHS. Compared with normoglycemia, diabetes was associated with an approximately three‐fold higher adjusted odds of SGHS (adjusted OR ≈ 2.8). Both HbA1c and fasting glucose were independently associated with a lower ratio and higher odds of SGHS after comprehensive multivariable adjustment. Insulin resistance also showed strong associations, particularly among individuals with normoglycemia, suggesting that metabolic disturbances are linked to eGFR biomarker discordance even before overt dysglycemia develops. Given the cross‐sectional design, these findings should be interpreted as associations rather than evidence of causality. However, whether the observed biomarker discordance reflects early kidney dysfunction, non‐GFR‐related determinants of cystatin C and creatinine, or both cannot be determined from the present study. The consistent associations across glycemic strata nevertheless suggest that metabolic dysregulation is reflected in the eGFR_cys_/eGFR_cr_‐ratio. Future longitudinal studies are needed to determine whether this biomarker discordance represents early kidney vulnerability or primarily reflects non‐GFR‐related determinants of cystatin C and creatinine.

### Clinical relevance

A low eGFR_cys_/eGFR_cr_‐ratio despite preserved creatinine‐based eGFR may identify individuals with early metabolic kidney vulnerability and increased cardiometabolic risk before conventional markers become abnormal. Although cystatin C may estimate GFR more accurately than creatinine in some clinical settings and has shown potential as an early marker of DKD [19], both markers are influenced by non‐GFR determinants [6]. Our findings extend these observations by demonstrating that the eGFR_cys_/eGFR_cr_‐ratio is already reduced in prediabetes, suggesting that eGFR biomarker discordance emerges early in the course of metabolic dysregulation. A key property of the eGFR_cys_/eGFR_cr_‐ratio is that it is largely independent of measured GFR [20, 21], making it a potentially valuable tool for identifying individuals with discordant filtration marker profiles despite preserved creatinine‐based kidney function. This aligns with recent findings in Type 2 diabetes where SGHS was associated with markedly increased mortality despite normal eGFR_cr_ [22]. Nevertheless, whether SGHS reflects true selective glomerular dysfunction, non‐GFR‐related filtration marker biology, or both remains uncertain. Longitudinal studies incorporating albuminuria and other markers of kidney injury and direct GFR measurements are needed to define the temporal sequence, biological basis, and prognostic significance of SGHS, and whether modification of metabolic risk factors alters its trajectory. Although the <0.7 threshold for defining a low eGFR_cys_/eGFR_cr_‐ratio originates from prior clinical studies, the optimal cutoff requires external validation.

### Potential mechanisms

Several mechanisms may contribute to our findings. Participants with impaired glycemic status had greater visceral adiposity, and previous studies have suggested that visceral fat accumulation is linked to higher circulating cystatin C levels [23], which may partly explain the increasing divergence between eGFR_cys_ and eGFR_cr_ across glycemic categories. Conversely, dysglycemia and insulin‐resistant states are often accompanied by reduced skeletal muscle mass [24], which lowers creatinine generation and may further amplify differences between eGFR estimates. Together, these mechanisms may contribute to a lower eGFR_cys_/eGFR_cr_‐ratio independent of true GFR. Previous studies have shown that insulin resistance and other cardiometabolic risk factors are associated with lower eGFR_cys_ and higher eGFR_cr_ despite similar measured GFR [25], supporting an important role for non‐GFR determinants of both filtration markers. Similarly, Mathisen et al. demonstrated that smoking was associated with higher measured GFR and that this hyperfiltration was more closely reflected by creatinine‐based than cystatin C‐based estimates [26]. Collectively, these findings suggest that discordance between creatinine‐ and cystatin C‐based eGFR may arise through mechanisms unrelated to selective glomerular dysfunction, warranting caution when interpreting a low eGFR_cys_/eGFR_cr_‐ratio as evidence of a specific renal process. Because visceral adiposity has been proposed as an important determinant of cystatin C concentrations, we examined whether the observed associations could be explained by differences in body composition. Adjustment for waist‐to‐height ratio, a validated indicator of visceral adiposity [27], did not materially alter the associations, suggesting that adiposity alone is unlikely to explain the findings. Cystatin C has been proposed to be affected by inflammation [28]. Although CRP was independently associated with both outcomes, additional adjustment resulted in only minimal attenuation of the associations, arguing against systemic inflammation as the sole explanation for the observed biomarker discordance. Furthermore, sensitivity analyses excluding participants with prevalent CVD and COPD yielded materially unchanged results, suggesting that these comorbidities are unlikely to explain the observed associations. Residual confounding from unmeasured non‐GFR determinants, including hormonal factors, comorbidity burden, smoking‐related effects, and vascular aging, cannot be excluded. Future studies incorporating directly measured GFR and longitudinal follow‐up are needed to clarify the relative contribution of kidney and non‐kidney mechanisms to the declining eGFR_cys_/eGFR_cr_‐ratio.

In sensitivity analyses modeling creatinine and cystatin C separately, dysglycemia demonstrated opposing associations with the two markers: Higher HbA1c and fasting glucose were associated with lower creatinine concentrations but higher cystatin C levels. This bidirectional pattern suggests that the widening biomarker gap reflects parallel changes in both markers rather than changes in a single biomarker, reinforcing that the ratio captures a physiologically meaningful signal.

Smoking, which was more prevalent in prediabetes and diabetes, also emerged as a strong determinant of both a lower eGFR_cys_/eGFR_cr_‐ratio and SGHS. Although its impact on cystatin C remains debated, smoking is known to induce oxidative stress, endothelial injury, and glomerular hyperfiltration [29], mechanisms that may exacerbate glycemia‐related vulnerability of the glomerular barrier. However, adjustment for smoking did not alter the observed associations.

Hyperglycemia may exert similar endothelial and inflammatory effects as smoking [30], potentially creating a synergistic burden on the glomerular filtration barrier. Additional mechanisms include promotion of glomerular basement membrane (GBM) thickening through increased extracellular matrix accumulation and reduced degradation [31]. Prior biopsy data have demonstrated that a lower eGFR_cys_/eGFR_cr_‐ratio correlates with GBM thickening [10]. Altered intraglomerular hemodynamics may also contribute, as early diabetic hyperfiltration increases capillary pressure, promoting podocyte injury and GBM expansion [32, 33]. However, early dysglycemia is also frequently associated with increased measured GFR [34], and estimating equations may not fully capture these dynamics. Accordingly, a declining eGFR_cys_/eGFR_cr_‐ratio may reflect both early glomerular alterations and non‐GFR‐related determinants, rather than selective hypofiltration alone. Studies incorporating measured GFR and longitudinal follow‐up are needed to disentangle these mechanisms.

Insulin resistance was associated with a lower eGFR_cys_/eGFR_cr_‐ratio across all glycemic strata, whereas its association with SGHS was strongest in normoglycemia and prediabetes and was not statistically significant in diabetes. It should be acknowledged that HOMA‐IR is less accurate in established dysglycemia [35]. This attenuation may reflect the reduced precision of HOMA‐IR in diabetes, greater heterogeneity in insulin secretion and treatment, or a stronger role of insulin resistance during earlier stages of metabolic dysfunction. This aligns with evidence linking insulin resistance to endothelial dysfunction, podocyte injury, and altered glomerular hemodynamics [36]. Early studies have also reported higher eGFR_cr_ in insulin‐resistant states, likely reflecting initial glomerular hyperfiltration [37]. Population‐based studies with measured GFR further suggest that hyperfiltration is more closely related to fasting glucose [38], indicating that hyperglycemia and insulin resistance represent interconnected processes. Notably, the difference in HOMA‐IR in our subjects depending on glycemic status was mainly driven by greater variance in fasting insulin levels. Given the widespread expression of insulin receptors in renal tissue, emerging evidence suggests that insulin itself may modulate glomerular structure and function, including endothelial integrity and podocyte signaling [39]. Taken together, these observations indicate that insulin resistance and hyperinsulinemia may contribute to eGFR biomarker discordance through a combination of glomerular and systemic metabolic pathways, potentially linking metabolic dysregulation to the development of a low eGFR_cys_/eGFR_cr_‐ratio. Although residual confounding from non‐GFR determinants of cystatin C cannot be excluded [6, 40], the consistency of associations across glycemic strata, alternative estimating equations, CRP‐adjusted models, and multiple sensitivity analyses argues against confounding as the sole explanation. Future studies incorporating direct GFR measurements will be required to determine the relative contributions of selective glomerular dysfunction and non‐kidney biomarker determinants to the declining eGFR_cys_/eGFR_cr_‐ratio.

### Strengths

Strengths include the large, well‐characterized, population‐based cohort with standardized measurements of both creatinine and cystatin C, enabling consistent assessment of discrepancies between creatinine‐ and cystatin C‐based estimates of GFR. The multicenter design enhances generalizability, and the comprehensive phenotyping allowed exploration of multiple metabolic and clinical pathways. The use of multiple estimating equations and extensive sensitivity analyses strengthened the robustness of our findings, with consistent associations observed across analytical approaches.

### Limitations

Several limitations should be acknowledged. The cross‐sectional design precludes causal inference, and longitudinal studies are required to determine whether selective glomerular hypofiltration predicts progression of glucometabolic disturbances or DKD. The absence of albuminuria data limits assessment of whether SGHS precedes, parallels, or adds prognostic information beyond established markers of kidney injury. Reliance on a single fasting glucose measurement may have introduced some misclassification, and the lack of oral glucose tolerance testing limited detection of impaired glucose tolerance. Minor analytical variation across study sites is possible but was partly addressed by adjustment for study site in all models. Residual confounding cannot be fully excluded despite comprehensive multivariable adjustment. Direct measures of body composition, frailty, and muscle mass were unavailable. As creatinine and cystatin C are influenced by non‐GFR determinants, including skeletal muscle mass, part of the observed biomarker discordance may, therefore, reflect body composition rather than altered glomerular filtration. Similarly, the absence of directly measured GFR precludes distinction between kidney and non‐kidney mechanisms underlying a low eGFR_cys_/eGFR_cr_‐ratio. Although analyses were restricted to participants with complete covariate data, only 4.0% of participants were excluded because of missingness. Comparison of included and excluded participants demonstrated largely similar distributions of age, glycemic measures, and the eGFR_cys_/eGFR_cr_‐ratio. Excluded participants were somewhat more likely to be male and had modestly higher BMI and SBP (Table S15). Because these variables were included in multivariable adjustment models, substantial selection bias due to missing covariate data appears unlikely.

An additional methodological consideration is the use of the CKD‐EPI 2021 equation. Although increasingly adopted due to its race‐free formulation and widespread international use, its performance in European populations remains less extensively studied than in North American cohorts. In addition, some studies have reported small systematic differences compared with previous equations [41]. To address this potential limitation and reduce reliance on a single estimating equation, we performed sensitivity analyses using the LMrev creatinine equation and the CAPA cystatin C equation, both of which are well established in Scandinavian populations. The overall findings remained essentially unchanged across equations, supporting the robustness of the observed associations. Finally, the participation rate in the SCAPIS was approximately 50%, which may limit the representativeness of the study population. However, previous evaluations of the SCAPIS cohort have suggested that selective participation primarily affects prevalence estimates, whereas associations between risk factors are considerably less influenced after accounting for demographic characteristics [42]. Accordingly, although some degree of selection bias cannot be excluded, the internal validity of the observed associations is likely to be preserved.

## Conclusions

In summary, dysglycemia and insulin resistance were associated with a lower eGFR_cys_/eGFR_cr_‐ratio and higher prevalence of SGHS across the full glycemic spectrum. These findings are consistent with two non‐mutually exclusive interpretations: early metabolic kidney vulnerability with altered glomerular permselectivity, and non‐GFR‐related metabolic effects on both biomarkers. The present cross‐sectional data cannot resolve their relative contributions. The eGFR_cys_/eGFR_cr_‐ratio may nonetheless serve as an integrative marker identifying individuals at elevated cardiometabolic risk despite normal creatinine‐based kidney function. Prospective studies incorporating direct GFR measurements and albuminuria are required to establish temporal sequence, prognostic value, and whether metabolic risk modification alters the trajectory of biomarker discordance.

## Author contributions

Christopher Nilsson contributed to the study design, performed the analyses, and drafted the manuscript. Agne Laucyte‐Cibulskiene, Martin Magnusson, Marcus A. Ohlsson, Anders Christensson, and Amra Jujic contributed to the study design and data interpretation and critically revised the manuscript draft, with Amra Jujic also preparing the figures. Gregor Guron, Maria K. Svensson, Maria Weiner, P. Andreas Jonsson, Johan Ärnlöv, Gunnar Engström, Anders Grubb, and Anders Larsson critically revised the manuscript. All authors read and approved the final version of the manuscript.

## Conflict of interest statement

The authors declare no conflicts of interest.

## Funding information

C.N. was supported by grants from Skane University Hospital. MM was supported by grants from the Medical Faculty of Lund University, Skåne University Hospital, the Crafoord Foundation, the Ernhold Lundstroms Research Foundation, the Region Skane, the Hulda and Conrad Mossfelt Foundation, the Southwest Skanes Diabetes Foundation, the Kockska Foundation, the Research Funds of Region Skåne, the Swedish Heart and Lung Foundation (2024‐0979), the Swedish Research Council (2022‐00973), and the Wallenberg Center for Molecular Medicine, Lund University, and Lund University. A.C. was supported by grants from the Swedish Kidney Foundation, Skåne University Hospital Research Fund, the Research and Development Council of Region Skåne, Sweden, and the Medical Faculty of Lund University. A.L.C. was supported by the Lund University (ALF young investigator grant), the Swedish Kidney Foundation, and the Skåne University Hospital Research Fund.

## Supporting information




**Table S1**: The association between HbA1c and the eGFR_cys_/eGFR_cr_‐ratio and SGHS status in all stepwise adjusted regression models.
**Table S2**: The association between fasting glucose and the eGFR_cys_/eGFR_cr_‐ratio and SGHS status in all stepwise adjusted regression models.
**Table S3**: The association between HbA1c and the eGFR_cys_/eGFR_cr_‐ratio and SGHS status in fully adjusted regression models. Sensitivity analysis based on the CAPA and LMrev equations.
**Table S4**: The association between fasting glucose and the eGFR_cys_/eGFR_cr_‐ratio and SGHS status in fully adjusted regression models. Sensitivity analysis based on the CAPA and LMrev equations.
**Table S5**: HOMA‐IR and its association with SGHS and the eGFR_cys_/eGFR_cr_‐ratio stratified by glycemic status. Sensitivity analysis based on the CAPA and LMrev equations.
**Table S6**: Sensitivity analysis: Insulin and its association with SGHS and the eGFR_cys_/eGFR_cr_‐ratio (CKD‐EPI equations) stratified by glycemic status.
**Table S7**: Sensitivity analysis: The association between HbA1c and creatinine, respectively, cystatin C in fully adjusted regression models.
**Table S8**: Sensitivity analysis: The association between fasting glucose and creatinine, respectively, cystatin C in fully adjusted regression models.
**Table S9**: Sensitivity analysis: The association between HbA1c and the eGFR_cys_/eGFR_cr_‐ratio and SGHS status in fully adjusted regression models in participants without CVD and COPD.
**Table S10**: Sensitivity analysis: The association between fasting glucose and the eGFR_cys_/eGFR_cr_‐ratio and SGHS status in fully adjusted regression models in participants without CVD and COPD.
**Table S11**: Sensitivity analysis: The association between HbA1c and the eGFR_cys_/eGFR_cr_‐ratio and SGHS status in fully adjusted regression models when excluding eGFR_cr‐cys_ as adjustment variable.
**Table S12**: Sensitivity analysis: The association between fasting glucose and the eGFR_cys_/eGFR_cr_‐ratio and SGHS status in fully adjusted regression models when excluding eGFR_cr‐cys_ as adjustment variable.
**Table S13**: Sensitivity analysis: The association between HbA1c and the eGFR_cys_/eGFR_cr_‐ratio and SGHS status in fully adjusted regression models, including CRP.
**Table S14**: Sensitivity analysis: The association between fasting glucose and the eGFR_cys_/eGFR_cr_‐ratio and SGHS status in fully adjusted regression models, including CRP.
**Table S15**: Characteristics of excluded participants in comparison to included participants.
