# Supplementary material for: Selective glomerular hypofiltration is associated with glucometabolic disturbances in the general population
Source: J Intern Med. 2026 Aug 12;300(4):499–512. doi: 10.1111/joim.70148 (PMC13569112; doi:10.1111/joim.70148)
Supplement: Supplementary file 1 [file JOIM-300-499-s001.docx]

**Supplement**

Supplementary table 1. The association between HbA1c with the eGFR_cys_/eGFR_cr_-ratio and SGHS status in all stepwise adjusted regression models.

|  | **eGFR_cys_/eGFR_cr_-ratio based on the CKD-EPI equations** | | | **SGHS (eGFR_cys_/eGFR_cr_-ratio <0.7) based on the CKD-EPI equation** | | |
| --- | --- | --- | --- | --- | --- | --- |
|  | β | 95% CI | P-value | OR | 95% CI | P-value |
| ***Model 1*** |  |  |  |  |  |  |
| Age | -0.033 | -0.038 to -0.028 | <0.001 | 1.185 | 1.062 to 1.322 | 0.002 |
| Male sex | -0.015 | -0.019 to -0.011 | <0.001 | 1.035 | 0.944 to 1.135 | 0.464 |
| eGFR | 0.005 | 0.005 to 0.005 | <0.001 | 0.921 | 0.918 to 0.925 | <0.001 |
|  |  |  |  |  |  |  |
| ***Model 2*** |  |  |  |  |  |  |
| Age | -0.027 | -0.032 to -0.023 | <0.001 | 1.098 | 0.983 to 1.227 | 0.099 |
| Male sex | -0.013 | -0.017 to -0.009 | <0.001 | 1.077 | 0.981 to 1.182 | 0.119 |
| eGFR | 0.005 | 0.005 to 0.005 | <0.001 | 0.922 | 0.918 to 0.925 | <0.001 |
| HbA1c | -0.031 | -0.034 to -0.028 | <0.001 | 1.514 | 1.429 to 1.605 | <0.001 |
|  |  |  |  |  |  |  |
| ***Model 3*** |  |  |  |  |  |  |
| Age | -0.026 | -0.031 to -0.022 | <0.001 | 1.090 | 0.971 to 1.224 | 0.143 |
| Male sex | -0.007 | -0.011 to -0.003 | <0.001 | 1.071 | 0.973 to 1.178 | 0.162 |
| eGFR | 0.004 | 0.004 to 0.004 | <0.001 | 0.927 | 0.923 to 0.930 | <0.001 |
| HbA1c | -0.015 | -0.018 to -0.012 | <0.001 | 1.265 | 1.188 to 1.348 | <0.001 |
| BMI | -0.008 | -0.008 to -0.008 | <0.001 | 1.105 | 1.094 to 1.115 | <0.001 |
| Smoking | -0.017 | -0.019 to -0.016 | <0.001 | 1.287 | 1.245 to 1.330 | <0.001 |
|  |  |  |  |  |  |  |
| ***Model 4*** |  |  |  |  |  |  |
| Age | -0.023 | -0.028 to -0.018 | <0.001 | 1.062 | 0.944 to 1.196 | 0.317 |
| Male sex | -0.006 | -0.010 to -0.002 | 0.003 | 1.088 | 0.987 to 1.198 | 0.089 |
| eGFR | 0.004 | 0.004 to 0.004 | <0.001 | 0.927 | 0.923 to 0.931 | <0.001 |
| HbA1c | -0.015 | -0.018 to -0.011 | <0.001 | 1.261 | 1.183 to 1.344 | <0.001 |
| BMI | -0.008 | -0.008 to -0.007 | <0.001 | 1.102 | 1.091 to 1.113 | <0.001 |
| Smoking | -0.017 | -0.019 to -0.016 | <0.001 | 1.286 | 1.245 to 1.330 | <0.001 |
| SBP | -0.001 | -0.001 to -0.001 | <0.001 | 1.003 | 1.000 to 1.006 | 0.028 |
| Antihypertensive treatment | -0.009 | -0.014 to -0.004 | <0.001 | 0.985 | 0.880 to 1.103 | 0.798 |
|  |  |  |  |  |  |  |
| ***Fully adjusted model 5*** |  |  |  |  |  |  |
| Age | −0.024 | -0.028 to -0.019 | <0.001 | 1.080 | 0.959 to 1.216 | 0.205 |
| Male sex | -0.006 | -0.010 to -0.002 | 0.002 | 1.093 | 0.992 to 1.204 | 0.076 |
| eGFR | 0.004 | 0.004 to 0.004 | <0.001 | 0.926 | 0.923 to 0.930 | <0.001 |
| HbA1c | -0.015 | -0.018 to -0.012 | <0.001 | 1.265 | 1.186 to 1.349 | <0.001 |
| BMI | -0.008 | -0.008 to -0.007 | <0.001 | 1.102 | 1.091 to 1.113 | <0.001 |
| Smoking | -0.017 | -0.019 to -0.016 | <0.001 | 1.291 | 1.248 to 1.334 | <0.001 |
| SBP | -0.001 | -0.001 to -0.001 | <0.001 | 1.004 | 1.001 to 1.007 | 0.017 |
| Antihypertensive treatment | -0.010 | -0.015 to -0.005 | <0.001 | 0.968 | 0.860 to 1.089 | 0.584 |
| LDL-C | 0.003 | 0.001 to 0.005 | 0.008 | 0.892 | 0.847 to 0.940 | <0.001 |
| Lipid-lowering treatment | 0.012 | 0.004 to 0.020 | 0.003 | 1.327 | 1.108 to 1.589 | 0.002 |

Abbreviations: BMI, body mass index; CI, confidence interval; CKD-EPI, Chronic Kidney Disease Epidemiology Collaboration; eGFR, estimated glomerular filtration rate; LDL-C, low-density lipoprotein cholesterol; OR, odds ratio; SBP, systolic blood pressure; SGHS, selective glomerular hypofiltration syndrome.

Regression coefficients (β) represent the change in the eGFR_cys_/eGFR_cr_-ratio per unit increase in the exposure. Odds ratios represent the odds of SGHS (eGFR_cys_/eGFR_cr_-ratio <0.7). eGFR denotes eGFR_-cr-cys_ (CKD-EPI 2021). Age and HbA1c were modelled per 10 units, BMI per 1 kg/m², smoking as log-transformed pack-years, SBP per 1 mmHg, and LDL-C per mmol/L. Antihypertensive and lipid-lowering treatments were modelled as binary variables. Study site was included as a fixed effect in all models.

Supplementary table 2. The association between fasting glucose with the eGFR_cys_/eGFR_cr_-ratio and SGHS status in all stepwise adjusted regression models.

|  | **eGFR_cys_/eGFR_cr_-ratio based on the CKD-EPI equations** | | | **SGHS (eGFR_cys_/eGFR_cr_-ratio <0.7) based on the CKD-EPI equation** | | |
| --- | --- | --- | --- | --- | --- | --- |
|  | β | 95% CI | P-value | OR | 95% CI | P-value |
| ***Model 1*** |  |  |  |  |  |  |
| Age | -0.033 | -0.038 to -0.029 | <0.001 | 1.185 | 1.062 to 1.322 | 0.002 |
| Male sex | -0.014 | -0.018 to -0.011 | <0.001 | 1.035 | 0.944 to 1.135 | 0.464 |
| eGFR | 0.005 | 0.005 to 0.005 | <0.001 | 0.921 | 0.917 to 0.924 | <0.001 |
|  |  |  |  |  |  |  |
| ***Model 2*** |  |  |  |  |  |  |
| Age | -0.028 | -0.032 to -0.023 | <0.001 | 1.114 | 0.997 to 1.244 | 0.057 |
| Male sex | -0.010 | -0.014 to -0.006 | <0.001 | 1.101 | 1.002 to 1.209 | 0.045 |
| eGFR | 0.005 | 0.005 to 0.005 | <0.001 | 0.921 | 0.917 to 0.924 | <0.001 |
| Glucose | -0.018 | -0.020 to -0.016 | <0.001 | 1.258 | 1.217 to 1.301 | <0.001 |
|  |  |  |  |  |  |  |
| ***Model 3*** |  |  |  |  |  |  |
| Age | -0.027 | -0.031 to -0.022 | <0.001 | 1.096 | 0.977 to 1.230 | 0.118 |
| Male sex | -0.006 | -0.010 to -0.002 | 0.002 | 1.089 | 0.997 to 1.184 | 0.136 |
| eGFR | 0.004 | 0.004 to 0.004 | <0.001 | 0.926 | 0.923 to 0.930 | <0.001 |
| Glucose | -0.009 | -0.011 to -0.007 | <0.001 | 1.135 | 1.094 to 1.177 | <0.001 |
| BMI | -0.008 | -0.008 to -0.007 | <0.001 | 1.104 | 1.094 to 1.115 | <0.001 |
| Smoking | -0.017 | -0.019 to -0.016 | <0.001 | 1.291 | 1.249 to 1.335 | <0.001 |
|  |  |  |  |  |  |  |
| ***Model 4*** |  |  |  |  |  |  |
| Age | -0.024 | -0.028 to -0.019 | <0.001 | 1.068 | 0.949 to 1.202 | 0.272 |
| Male sex | -0.005 | -0.009 to -0.001 | 0.014 | 1.092 | 0.992 to 1.204 | 0.074 |
| eGFR | 0.004 | 0.004 to 0.004 | <0.001 | 0.926 | 0.923 to 0.930 | <0.001 |
| Glucose | -0.008 | -0.008 to -0.007 | <0.001 | 1.132 | 1.091 to 1.175 | <0.001 |
| BMI | -0.008 | -0.008 to -0.007 | <0.001 | 1.102 | 1.091 to 1.113 | <0.001 |
| Smoking | -0.017 | -0.019 to -0.016 | <0.001 | 1.291 | 1.249 to 1.335 | <0.001 |
| SBP | -0.001 | -0.001 to -0.001 | <0.001 | 1.003 | 1.000 to 1.006 | 0.028 |
| Antihypertensive treatment | -0.008 | -0.013 to -0.003 | 0.001 | 0.982 | 0.882 to 1.106 | 0.832 |
|  |  |  |  |  |  |  |
| ***Fully adjusted model 5*** |  |  |  |  |  |  |
| Age | -0.024 | -0.029 to -0.020 | <0.001 | 1.086 | 0.965 to 1.224 | 0.172 |
| Male sex | -0.005 | -0.009 to -0.001 | 0.010 | 1.094 | 0.993 to 1.206 | 0.069 |
| eGFR | 0.004 | 0.004 to 0.004 | <0.001 | 0.926 | 0.922 to 0.930 | <0.001 |
| Glucose | -0.008 | -0.010 to -0.006 | <0.001 | 1.124 | 1.081 to 1.168 | <0.001 |
| BMI | -0.008 | -0.008 to -0.007 | <0.001 | 1.103 | 1.092 to 1.114 | <0.001 |
| Smoking | -0.017 | -0.019 to -0.016 | <0.001 | 1.295 | 1.253 to 1.339 | <0.001 |
| SBP | -0.001 | -0.001 to -0.001 | <0.001 | 1.004 | 1.001 to 1.007 | 0.018 |
| Antihypertensive treatment | -0.010 | -0.015 to -0.005 | <0.001 | 0.970 | 0.862 to 1.091 | 0.606 |
| LDL-C | 0.001 | -0.001 to 0.004 | 0.176 | 0.908 | 0.861 to 0.957 | <0.001 |
| Lipid-lowering treatment | 0.009 | 0.002 to 0.017 | 0.018 | 1.268 | 1.061 to 1.517 | 0.009 |

Abbreviations: BMI, body mass index; CI, confidence interval; CKD-EPI, Chronic Kidney Disease Epidemiology Collaboration; eGFR, estimated glomerular filtration rate; LDL-C, low-density lipoprotein cholesterol; OR, odds ratio; SBP, systolic blood pressure; SGHS, selective glomerular hypofiltration syndrome.

Regression coefficients (β) represent the change in the eGFR_cys_/eGFR_cr_-ratio per 1 mmol/L increase in fasting glucose. Odds ratios represent the odds of SGHS (eGFR_cys_/eGFR_cr_-ratio <0.7). eGFR denotes eGFR_-cr-cys_ (CKD-EPI 2021). Age was modelled per 10 years, BMI per 1 kg/m², smoking as log-transformed pack-years, SBP per 1 mmHg, and LDL-C per mmol/L. Antihypertensive and lipid-lowering treatments were modelled as binary variables. Study site was included as a fixed effect in all models.

Supplementary table 3. The association between HbA1c with the eGFR_cys_/eGFR_cr_-ratio and SGHS status in fully adjusted regression models. Sensitivity analysis based on the CAPA and LMrev equations.

|  | **eGFR_cys_/eGFR_cr_-ratio based on the CAPA and LMrev equations** | | | **SGHS (eGFR_cys_/eGFR_cr_-ratio <0.7) based on the CAPA and LMrev equations** | | |
| --- | --- | --- | --- | --- | --- | --- |
| ***Fully adjusted model (5)*** | β | 95% CI | P-value | OR | 95% CI | P-value |
| Age | 0.023 | 0.018 to 0.028 | <0.001 | 0.650 | 0.515 to 0.822 | <0.001 |
| Male sex | -0.050 | -0.054 to -0.046 | <0.001 | 0.678 | 0.559 to 0.823 | <0.001 |
| eGFR | 0.006 | 0.006 to 0.006 | <0.001 | 0.921 | 0.914 to 0.927 | <0.001 |
| HbA1c | -0.018 | -0.021 to -0.014 | <0.001 | 1.264 | 1.137 to 1.405 | <0.001 |
| BMI | -0.008 | -0.008 to -0.007 | <0.001 | 1.118 | 1.099 to 1.137 | <0.001 |
| Smoking | -0.018 | -0.020 to -0.017 | <0.001 | 1.385 | 1.297 to 1.479 | <0.001 |
| SBP | -0.001 | -0.001 to -0.001 | <0.001 | 1.001 | 0.995 to 1.007 | 0.776 |
| Antihypertensive treatment | -0.008 | -0.014 to -0.003 | 0.003 | 1.046 | 0.834 to 1.313 | 0.696 |
| LDL-C | 0.003 | 0.001 to 0.005 | 0.015 | 0.771 | 0.695 to 0.855 | <0.001 |
| Lipid-lowering treatment | 0.010 | 0.002 to 0.019 | 0.016 | 1.638 | 1.170 to 2.293 | 0.004^[1]^ |

Abbreviations: BMI, body mass index; CAPA, Caucasian, Asian, Pediatric, and Adult cystatin C–based estimating equation; CI, confidence interval; eGFR, estimated glomerular filtration rate; LDL-C, low-density lipoprotein cholesterol; LMrev, revised Lund–Malmö creatinine-based estimating equation; OR, odds ratio; SBP, systolic blood pressure; SGHS, selective glomerular hypofiltration syndrome.

eGFR_cys_ was estimated using the CAPA equation and eGFR^cr^ using the revised Lund–Malmö equation. Regression coefficients (β) represent the change in the eGFR_cys_/eGFR_cr_-ratio per 10 mmol/mol increase in HbA1c. Odds ratios represent the odds of SGHS (eGFR_cys_/eGFR_cr_-ratio <0.7). Age was modeled per 10 years; HbA1c per 10 mmol/mol; BMI per 1 kg/m²; smoking exposure as ln(pack-years + 1); SBP per 1 mmHg; LDL-C per 1 mmol/L; antihypertensive and lipid-lowering treatment as binary variables (yes/no). Fully adjusted according to Model 5. Study site was included as a fixed effect.

Supplementary table 4. The association between fasting glucose with the eGFR_cys_/eGFR_cr_-ratio and SGHS status in fully adjusted regression models. Sensitivity analysis based on the CAPA and LMrev equations.

|  | **eGFR_cys_/eGFR_cr_-ratio based on the CAPA and LMrev equations** | | | **SGHS (eGFR_cys_/eGFR_cr_-ratio <0.7) based on the CAPA and LMrev equations** | | |
| --- | --- | --- | --- | --- | --- | --- |
| ***Fully adjusted model (5)*** | β | 95% CI | P-value | OR | 95% CI | P-value |
| Age | 0.023 | 0.018 to 0.028 | <0.001 | 0.646 | 0.511 to 0.817 | <0.001 |
| Male sex | -0.048 | -0.053 to -0.044 | <0.001 | 0.681 | 0.561 to 0.827 | <0.001 |
| eGFR | 0.006 | 0.006 to 0.006 | <0.001 | 0.920 | 0.913 to 0.926 | <0.001 |
| Glucose | -0.010 | -0.012 to -0.008 | <0.001 | 1.183 | 1.115 to 1.255 | <0.001 |
| BMI | -0.008 | -0.008 to -0.007 | <0.001 | 1.116 | 1.097 to 1.135 | <0.001 |
| Smoking | -0.018 | -0.020 to -0.017 | <0.001 | 1.389 | 1.300 to 1.483 | <0.001 |
| SBP | -0.001 | -0.001 to -0.001 | <0.001 | 1.000 | 0.995 to 1.006 | 0.898 |
| Antihypertensive treatment | -0.004 | -0.014 to -0.003 | 0.004 | 1.044 | 0.832 to 1.309 | 0.795 |
| LDL-C | 0.001 | -0.001 to 0.003 | 0.323 | 0.795 | 0.716 to 0.883 | <0.001 |
| Lipid-lowering treatment | 0.007 | -0.001 to 0.016 | 0.078 | 1.613 | 1.154 to 2.256 | 0.005 |

Abbreviations: BMI, body mass index; CAPA, Caucasian, Asian, Pediatric, and Adult cystatin C–based estimating equation; CI, confidence interval; eGFR, estimated glomerular filtration rate; LDL-C, low-density lipoprotein cholesterol; LMrev, revised Lund–Malmö creatinine-based estimating equation; OR, odds ratio; SBP, systolic blood pressure; SGHS, selective glomerular hypofiltration syndrome.

eGFR_cys_ was estimated using the CAPA equation and eGFR_cr_ using the revised Lund–Malmö equation. Regression coefficients (β) represent the change in the eGFR_cys_/eGFR_cr_-ratio per 1 mmol/L increase in fasting glucose. Odds ratios represent the odds of SGHS (eGFR_cys_/eGFR_cr_-ratio <0.7). Age was modeled per 10 years; BMI per 1 kg/m²; smoking exposure as ln(pack-years + 1); SBP per 1 mmHg; LDL-C per 1 mmol/L; antihypertensive and lipid-lowering treatment as binary variables (yes/no). Fully adjusted according to Model 5. Study site was included as a fixed effect.

Supplementary table 5. HOMA-IR and its association with SGHS and the eGFR_cys_/eGFR_cr_-ratio stratified by glycaemic status. Sensitivity analysis based on the CAPA and LMrev equations.

|  | **SGHS** | | |
| --- | --- | --- | --- |
|  | OR | 95% CI | P-value |
| Normoglycaemia | 1.392 | 1.062 to 1.825 | 0.016 |
| Prediabetes | 1.373 | 0.922 to 2.045 | 0.118 |
| Diabetes | 1.274 | 1.005 to 1.615 | 0.046 |
|  |  |  |  |
|  | **eGFR_cys_/eGFR_cr_-ratio** | | |
|  | β | 95% CI | P-value |
| Normoglycaemia | -0.041 | -0.046to -0.036 | <0.001 |
| Prediabetes | -0.044 | -0.054 to -0.033 | <0.001 |
| Diabetes | -0.014 | -0.024 to -0.004 | 0.004 |

Abbreviations: CAPA, Caucasian, Asian, Pediatric, and Adult cystatin C–based estimating equation; CI, confidence interval; eGFR, estimated glomerular filtration rate; HOMA-IR, homeostasis model assessment of insulin resistance; LMrev, revised Lund–Malmö creatinine-based estimating equation; OR, odds ratio; SGHS, selective glomerular hypofiltration syndrome.

HOMA-IR was log-transformed before analysis. Regression coefficients (β) represent the change in the eGFR_cys_/eGFR_cr_-ratio per unit increase in log-transformed HOMA-IR. Odds ratios represent the odds of SGHS (eGFR_cys_/eGFR_cr_-ratio <0.7). Fully adjusted according to Model 5. Study site was included as a fixed effect.

Supplementary table 6. Sensitivity analysis: Insulin and its association with SGHS and the eGFR_cys_/eGFR_cr_-ratio (CKD-EPI equations) stratified by glycaemic status.

|  | **SGHS** | | |
| --- | --- | --- | --- |
|  | OR | 95% CI | P-value |
| Normoglycaemia | 1.923 | 1.403 to 2.635 | <0.001 |
| Prediabetes | 2.446 | 1.432 to 4.178 | <0.001 |
| Diabetes | 1.354 | 0.855 to 2.145 | 0.196 |
|  |  |  |  |
|  | **eGFR_cys_/eGFR_cr_-ratio** | | |
|  | β | 95% CI | P-value |
| Normoglycaemia | -0.098 | -0.110 to -0.086 | <0.001 |
| Prediabetes | -0.097 | -0.120 to -0.074 | <0.001 |
| Diabetes | -0.028 | -0.051 to -0.005 | 0.018 |

Abbreviations: CI, confidence interval; CKD-EPI, Chronic Kidney Disease Epidemiology Collaboration; eGFR, estimated glomerular filtration rate; OR, odds ratio; SGHS, selective glomerular hypofiltration syndrome.

Fasting insulin was log-transformed before analysis. Regression coefficients (β) represent the change in the eGFR_cys_/eGFR_cr_-ratio per unit increase in log-transformed insulin; ORs represent odds of SGHS (defined as eGFR_cys_/eGFR_cr_-ratio <0.7). eGFR denotes eGFR_-cr-cys_ (CKD-EPI 2021). Fully adjusted according to Model 5. Study site was included as a fixed effect.

Supplementary table 7. Sensitivity analysis: The association between HbA1c and creatinine respectively cystatin C in fully adjusted regression models

|  | **Creatinine** | | | **Cystatin C** | | |
| --- | --- | --- | --- | --- | --- | --- |
| ***Fully adjusted model (5)*** | β | 95% CI | P-value | β | 95% CI | P-value |
| Age | -0.028 | -0.029 to -0.027 | <0.001 | -0.017 | -0.019 to -0.015 | <0.001 |
| Male sex | 0.104 | 0.103 to 0.105 | <0.001 | 0.059 | 0.057 to 0.061 | <0.001 |
| eGFR | -0.004 | -0.004 to -0.004 | <0.001 | -0.011 | -0.011 to -0.011 | <0.001 |
| HbA1c | -0.006 | -0.007 to -0.005 | <0.001 | 0.010 | 0.009 to 0.012 | <0.001 |
| BMI | -0.002 | -0.002 to -0.002 | <0.001 | 0.003 | 0.003 to 0.003 | <0.001 |
| Smoking | -0.005 | -0.006 to -0.005 | <0.001 | 0.008 | 0.007 to 0.008 | <0.001 |
| SBP | -0.001 | -0.001 to -0.001 | <0.001 | 0.001 | 0.001 to 0.001 | <0.001 |
| Antihypertensive treatment | -0.002 | -0.003 to -0.001 | 0.005 | 0.010 | 0.008 to 0.012 | <0.001 |
| LDL-C | 0.002 | 0.001 to 0.002 | <0.001 | -0.002 | -0.003 to -0.002 | <0.001 |
| Lipid-lowering treatment | 0.004 | 0.002 to 0.006 | <0.001 | -0.001 | -0.005 to 0.002 | 0.420 |

Abbreviations: BMI, body mass index; CI, confidence interval; eGFR, estimated glomerular filtration rate; LDL-C, low-density lipoprotein cholesterol; SBP, systolic blood pressure.

Models are fully adjusted (Model 5). eGFR denotes combined creatinine–cystatin C eGFR (eGFR_cr-cys_) estimated using the CKD-EPI 2021 equation. Study site was included as a fixed effect. Age was modeled per 10 years; HbA1c per 10 mmol/mol; BMI per 1 kg/m²; smoking exposure as ln(pack-years + 1); SBP per 1 mmHg; LDL-C per 1 mmol/L; antihypertensive and lipid-lowering treatment as binary variables (yes/no). Creatinine was log-transformed before analysis. Regression coefficients (β) represent the change in log-creatinine or cystatin C per unit increase in the exposure.

Supplementary table 8. Sensitivity analysis: The association between fasting glucose and creatinine respectively cystatin C in fully adjusted regression models

Abbreviations: BMI, body mass index; CI, confidence interval; eGFR, estimated glomerular filtration rate; LDL-C, low-density lipoprotein cholesterol; SBP, systolic blood pressure.

Models are fully adjusted (Model 5). eGFR denotes combined creatinine–cystatin C eGFR (eGFR_cr-cys_) estimated using the CKD-EPI 2021 equation. Study site was included as a fixed effect. Age was modeled per 10 years; fasting glucose per 1 mmol/L; BMI per 1 kg/m²; smoking exposure as ln(pack-years + 1); SBP per 1 mmHg; LDL-C per 1 mmol/L; antihypertensive and lipid-lowering treatment as binary variables (yes/no). Creatinine was log-transformed before analysis. Regression coefficients (β) represent the change in log-creatinine or cystatin C per unit increase in the exposure.

|  | **Creatinine** | | | **Cystatin C** | | |
| --- | --- | --- | --- | --- | --- | --- |
| ***Fully adjusted model (5)*** | β | 95% CI | P-value | β | 95% CI | P-value |
| Age | -0.028 | -0.030 to -0.027 | <0.001 | -0.016 | -0.019 to -0.014 | <0.001 |
| Male sex | 0.105 | 0.104 to 0.106 | <0.001 | 0.058 | 0.057 to 0.060 | <0.001 |
| eGFR | -0.004 | -0.004 to -0.004 | <0.001 | -0.011 | -0.011 to -0.011 | <0.001 |
| Glucose | -0.003 | -0.004 to -0.003 | <0.001 | 0.005 | 0.004 to 0.006 | <0.001 |
| BMI | -0.002 | -0.002 to -0.002 | <0.001 | 0.003 | 0.003 to 0.003 | <0.001 |
| Smoking | -0.005 | -0.006 to -0.005 | <0.001 | 0.008 | 0.007 to 0.008 | <0.001 |
| SBP | -0.001 | -0.001 to -0.001 | <0.001 | 0.001 | 0.001 to 0.001 | 0.003 |
| Antihypertensive treatment | -0.002 | -0.003 to -0.001 | 0.007 | 0.010 | 0.008 to 0.012 | <0.001 |
| LDL-C | 0.001 | 0.001 to 0.002 | <0.001 | -0.002 | -0.003 to -0.001 | <0.001 |
| Lipid-lowering treatment | 0.003 | 0.001 to 0.005 | 0.002 | 0.000 | -0.003 to 0.004 | 0.832 |

Supplementary table 9. Sensitivity analysis: The association between HbA1c with the eGFR_cys_/eGFR_cr_-ratio and SGHS status in fully adjusted regression models in participants without CVD and COPD.

|  | **eGFR_cys_/eGFR_cr_-ratio based on the CKD-EPI equations** | **SGHS (eGFR_cys_/eGFR_cr_-ratio <0.7) based on the CKD-EPI equation** |
| --- | --- | --- |

| ***Fully adjusted model 5*** | β | 95% CI | P-value | OR | 95% CI | P-value |
| --- | --- | --- | --- | --- | --- | --- |
| Age | -0.024 | -0.028 to -0.019 | <0.001 | 1.080 | 0.959 to 1.216 | 0.205 |
| Male sex | -0.006 | -0.010 to -0.002 | 0.002 | 1.093 | 0.992 to 1.204 | 0.073 |
| eGFR | 0.004 | 0.004 to 0.004 | <0.001 | 0.926 | 0.923 to 0.930 | <0.001 |
| HbA1c | -0.015 | -0.018 to -0.012 | <0.001 | 1.265 | 1.186 to 1.349 | <0.001 |
| BMI | -0.008 | -0.008 to -0.007 | <0.001 | 1.102 | 1.091 to 1.113 | <0.001 |
| Smoking | -0.017 | -0.019 to -0.016 | <0.001 | 1.291 | 1.248 to 1.334 | <0.001 |
| SBP | -0.001 | -0.001 to -0.001 | <0.001 | 1.004 | 1.001 to 1.007 | 0.017 |
| Antihypertensive treatment | -0.010 | -0.015 to -0.005 | <0.001 | 0.968 | 0.860 to 1.089 | 0.584 |
| LDL-C | 0.003 | 0.001 to 0.005 | 0.008 | 0.892 | 0.847 to 0.940 | <0.001 |
| Lipid-lowering treatment | 0.012 | 0.004 to 0.020 | 0.003 | 1.327 | 1.108 to 1.589 | 0.002 |

Abbreviations: BMI, body mass index; CI, confidence interval; CKD-EPI, Chronic Kidney Disease Epidemiology Collaboration; eGFR, estimated glomerular filtration rate; LDL-C, low-density lipoprotein cholesterol; OR, odds ratio; SBP, systolic blood pressure; SGHS, selective glomerular hypofiltration syndrome.

Regression coefficients (β) represent the change in the eGFR_cys_/eGFR_cr_-ratio per unit increase in the exposure. Odds ratios represent the odds of SGHS (eGFR_cys_/eGFR_cr_-ratio <0.7). eGFR denotes eGFR_-cr-cys_ (CKD-EPI 2021). Age and HbA1c were modelled per 10 units, BMI per 1 kg/m², smoking as log-transformed pack-years, SBP per 1 mmHg, and LDL-C per mmol/L. Antihypertensive and lipid-lowering treatments were modelled as binary variables. Study site was included as a fixed effect in all models.

Supplementary table 10. Sensitivity analysis: The association between fasting glucose with the eGFR_cys_/eGFR_cr_-ratio and SGHS status in fully adjusted regression models in participants without CVD and COPD.

| ***Fully adjusted model 5*** | β | 95% CI | P-value | OR | 95% CI | P-value |
| --- | --- | --- | --- | --- | --- | --- |
| Age | -0.024 | -0.029 to -0.020 | <0.001 | 1.086 | 0.965 to 1.223 | 0.172 |
| Male sex | -0.005 | -0.009 to -0.001 | 0.010 | 1.094 | 0.993 to 1.206 | 0.069 |
| eGFR | 0.004 | 0.004 to 0.004 | <0.001 | 0.926 | 0.922 to 0.930 | <0.001 |
| Glucose | -0.008 | -0.010 to -0.006 | <0.001 | 1.124 | 1.081 to 1.168 | <0.001 |
| BMI | -0.008 | -0.008 to -0.007 | <0.001 | 1.103 | 1.092 to 1.114 | <0.001 |
| Smoking | -0.017 | -0.019 to -0.016 | <0.001 | 1.295 | 1.253 to 1.339 | <0.001 |
| SBP | -0.001 | -0.001 to -0.001 | <0.001 | 1.004 | 1.001 to 1.007 | 0.018 |
| Antihypertensive treatment | -0.010 | -0.015 to -0.005 | <0.001 | 0.970 | 0.862 to 1.091 | 0.606 |
| LDL-C | 0.001 | -0.001 to 0.004 | 0.176 | 0.908 | 0.861 to 0.957 | <0.001 |
| Lipid-lowering treatment | 0.009 | 0.002 to 0.017 | 0.018 | 1.268 | 1.061 to 1.517 | 0.009 |

Abbreviations: BMI, body mass index; CI, confidence interval; CKD-EPI, Chronic Kidney Disease Epidemiology Collaboration; eGFR, estimated glomerular filtration rate; LDL-C, low-density lipoprotein cholesterol; OR, odds ratio; SBP, systolic blood pressure; SGHS, selective glomerular hypofiltration syndrome.

Regression coefficients (β) represent the change in the eGFR_cys_/eGFR_cr_-ratio per unit increase in the exposure. Odds ratios represent the odds of SGHS (eGFR_cys_/eGFR_cr_-ratio <0.7). eGFR denotes eGFR_-cr-cys_ (CKD-EPI 2021). Age was modelled per 10 units, BMI per 1 kg/m², smoking as log-transformed pack-years, fasting glucose per 1 mmol/L, SBP per 1 mmHg, and LDL-C per mmol/L. Antihypertensive and lipid-lowering treatments were modelled as binary variables. Study site was included as a fixed effect in all models.

Supplementary table 11. Sensitivity analysis: The association between HbA1c with the eGFR_cys_/eGFR_cr_-ratio and SGHS status in fully adjusted regression models when excluding eGFR_cr-cys_ as adjustment variable.

|  | **eGFR_cys_/eGFR_cr_-ratio based on the CKD-EPI equations** | **SGHS (eGFR_cys_/eGFR_cr_-ratio <0.7) based on the CKD-EPI equation** |
| --- | --- | --- |

| ***Fully adjusted model 5*** | β | 95% CI | P-value | OR | 95% CI | P-value |
| --- | --- | --- | --- | --- | --- | --- |
| Age | -0.052 | -0.057 to -0.048 | <0.001 | 1.741 | 1.559 to 1.945 | <0.001 |
| Male sex | 0.002 | -0.002 to 0.006 | 0.350 | 1.255 | 1.145 ti 1.375 | <0.001 |
| HbA1c | -0.014 | -0.017 to -0.010 | <0.001 | 1.202 | 1.136 to 1.272 | <0.001 |
| BMI | -0.008 | -0.008 to -0.007 | <0.001 | 1.131 | 1.120 to 1.142 | <0.001 |
| Smoking | -0.017 | -0.019 to -0.016 | <0.001 | 1.275 | 1.235 to 1.315 | <0.001 |
| SBP | -0.001 | -0.001 to -0.001 | 0.002 | 1.003 | 1.000 to 1.005 | 0.082 |
| Antihypertensive treatment | -0.020 | -0.026 to -0.015 | <0.001 | 0.785 | 0.703 to 0.876 | <0.001 |
| LDL-C | 0.001 | -0.002 to 0.002 | 0.836 | 0.939 | 0.893 to 0.986 | 0.012 |
| Lipid-lowering treatment | 0.012 | 0.004 to 0.021 | 0.003 | 1.252 | 1.059 to 1.480 | 0.008 |

Abbreviations: BMI, body mass index; CI, confidence interval; CKD-EPI, Chronic Kidney Disease Epidemiology Collaboration; eGFR, estimated glomerular filtration rate; LDL-C, low-density lipoprotein cholesterol; OR, odds ratio; SBP, systolic blood pressure; SGHS, selective glomerular hypofiltration syndrome.

Regression coefficients (β) represent the change in the eGFR_cys_/eGFR_cr_-ratio per unit increase in the exposure. Odds ratios represent the odds of SGHS (eGFR_cys_/eGFR_cr_-ratio <0.7). Age and HbA1c were modelled per 10 units, BMI per 1 kg/m², smoking as log-transformed pack-years, SBP per 1 mmHg, and LDL-C per mmol/L. Antihypertensive and lipid-lowering treatments were modelled as binary variables. Study site was included as a fixed effect in all models.

Supplementary table 12. Sensitivity analysis: The association between fasting glucose with the eGFR_cys_/eGFR_cr_-ratio and SGHS status in fully adjusted regression models when excluding eGFR_cr-cys_ as adjustment variable.

|  | **eGFR_cys_/eGFR_cr_-ratio based on the CKD-EPI equations** | **SGHS (eGFR_cys_/eGFR_cr_-ratio <0.7) based on the CKD-EPI equation** |
| --- | --- | --- |

| ***Fully adjusted model 5*** | β | 95% CI | P-value | OR | 95% CI | P-value |
| --- | --- | --- | --- | --- | --- | --- |
| Age | -0.054 | -0.058 to -0.049 | <0.001 | 1.764 | 1.580 to 1.970 | <0.001 |
| Male sex | 0.002 | -0.002 to 0.006 | 0.230 | 1.246 | 1.137 to 1.365 | <0.001 |
| Glucose | -0.004 | -0.006 to -0.002 | <0.001 | 1.060 | 1.023 to 1.098 | 0.001 |
| BMI | -0.010 | -0.010 to -0.009 | <0.001 | 1.134 | 1.123 to 1.144 | <0.001 |
| Smoking | -0.018 | -0.019 to -0.016 | <0.001 | 1.280 | 1.240 to 1.321 | <0.001 |
| SBP | -0.001 | -0.001 to -0.001 | 0.002 | 1.003 | 1.000 to 1.006 | 0.070 |
| Antihypertensive treatment | -0.020 | -0.026 to -0.015 | <0.001 | 0.781 | 0.700 to 0.871 | <0.001 |
| LDL-C | 0.001 | -0.003 to 0.002 | 0.768 | 0.943 | 0.897 to 0.991 | 0.021 |
| Lipid-lowering treatment | 0.009 | 0.001 to 0.017 | 0.032 | 1.189 | 1.007 to 1.402 | 0.041 |

Abbreviations: BMI, body mass index; CI, confidence interval; CKD-EPI, Chronic Kidney Disease Epidemiology Collaboration; eGFR, estimated glomerular filtration rate; LDL-C, low-density lipoprotein cholesterol; OR, odds ratio; SBP, systolic blood pressure; SGHS, selective glomerular hypofiltration syndrome.

Regression coefficients (β) represent the change in the eGFR_cys_/eGFR_cr_-ratio per unit increase in the exposure. Odds ratios represent the odds of SGHS (eGFR_cys_/eGFR_cr_-ratio <0.7). Age was modelled per 10 units, BMI per 1 kg/m², smoking as log-transformed pack-years, fasting glucose per 1 mmol/L, SBP per 1 mmHg, and LDL-C per mmol/L. Antihypertensive and lipid-lowering treatments were modelled as binary variables. Study site was included as a fixed effect in all models.

Supplementary table 13. Sensitivity analysis: The association between HbA1c with the eGFR_cys_/eGFR_cr_-ratio and SGHS status in fully adjusted regression models including CRP.

|  | **eGFR_cys_/eGFR_cr_-ratio based on the CKD-EPI equations** | **SGHS (eGFR_cys_/eGFR_cr_-ratio <0.7) based on the CKD-EPI equation** |
| --- | --- | --- |

| ***Fully adjusted model 5*** | β | 95% CI | P-value | OR | 95% CI | P-value |
| --- | --- | --- | --- | --- | --- | --- |
| Age | -0.023 | -0.027 to -0.018 | <0.001 | 1.083 | 0.961 to 1.220 | 0.189 |
| Male sex | -0.009 | -0.013 to -0.005 | <0.001 | 1.062 | 0.963 to 1.170 | 0.230 |
| eGFR | 0.004 | 0.004 to 0.004 | <0.001 | 0.929 | 0.925 to 0.932 | <0.001 |
| Hba1C | -0.012 | -0.015 to -0.008 | <0.001 | 1.215 | 1.138 to 1.297 | <0.001 |
| BMI | -0.006 | -0.006 to -0.005 | <0.001 | 1.080 | 1.068 to 1.091 | <0.001 |
| Smoking | -0.016 | -0.018 to -0.015 | <0.001 | 1.273 | 1.231 to 1.316 | <0.001 |
| SBP | -0.001 | -0.001 to -0.001 | <0.001 | 1.003 | 1.000 to 1.006 | 0.052 |
| Antihypertensive treatment | -0.009 | -0.014 to -0.004 | <0.001 | 0.974 | 0.865 to 1.096 | 0.661 |
| LDL-C | 0.003 | 0.002 to 0.004 | <0.001 | 0.884 | 0.838 to 0.931 | <0.001 |
| Lipid-lowering treatment | 0.005 | -0.003 to 0.013 | 0.235 | 1.221 | 1.018 to 1.464 | 0.031 |
| CRP | -0.064 | -0.070 to -0.059 | <0.001 | 2.107 | 1.854 to 2.394 | <0.001 |

Abbreviations: BMI, body mass index; CI, confidence interval; CKD-EPI, Chronic Kidney Disease Epidemiology Collaboration; CRP, C-reactive protein; eGFR, estimated glomerular filtration rate; LDL-C, low-density lipoprotein cholesterol; OR, odds ratio; SBP, systolic blood pressure; SGHS, selective glomerular hypofiltration syndrome.

Regression coefficients (β) represent the change in the eGFR_cys_/eGFR_cr_-ratio per unit increase in the exposure. Odds ratios represent the odds of SGHS (eGFR_cys_/eGFR_cr_-ratio <0.7).. Age and HbA1c were modelled per 10 units, BMI per 1 kg/m², smoking as log-transformed pack-years, SBP per 1 mmHg, and LDL-C per mmol/L. Antihypertensive and lipid-lowering treatments were modelled as binary variables. Study site was included as a fixed effect in all models.

Supplementary table 14. Sensitivity analysis: The association between fasting glucose with the eGFR_cys_/eGFR_cr_-ratio and SGHS status in fully adjusted regression models including CRP.

|  | **eGFR_cys_/eGFR_cr_-ratio based on the CKD-EPI equations** | **SGHS (eGFR_cys_/eGFR_cr_-ratio <0.7) based on the CKD-EPI equation** |
| --- | --- | --- |

| ***Fully adjusted model 5*** | β | 95% CI | P-value | OR | 95% CI | P-value |
| --- | --- | --- | --- | --- | --- | --- |
| Age | -0.023 | -0.028 to -0.019 | <0.001 | 1.087 | 0.965 to 1.225 | 0.168 |
| Male sex | -0.008 | -0.012 to 0.004 | <0.001 | 1.061 | 0.962 to 1.170 | 0.237 |
| eGFR | 0.004 | 0.004 to 0.004 | <0.001 | 0.928 | 0.925 to 0.932 | <0.001 |
| Glucose | -0.007 | -0.009 to -0.005 | <0.001 | 1.104 | 1.062 to 1.148 | <0.001 |
| BMI | -0.006 | -0.006 to -0.005 | <0.001 | 1.079 | 1.068 to 1.091 | <0.001 |
| Smoking | -0.016 | -0.018 to -0.015 | <0.001 | 1.277 | 1.235 to 1.320 | <0.001 |
| SBP | -0.001 | -0.001 to -0.001 | 0.001 | 1.003 | 1.000 to 1.006 | 0.056 |
| Antihypertensive treatment | -0.009 | -0.014 to -0.004 | <0.001 | 0.977 | 0.868 to 1.099 | 0.694 |
| LDL-C | 0.002 | 0.000 to 0.004 | 0.091 | 0.897 | 0.851 to 0.946 | <0.001 |
| Lipid-lowering treatment | 0.003 | -0.005 to 0.011 | 0.457 | 1.175 | 0.982 to 1.407 | 0.079 |
| CRP | -0.065 | -0.070 to -0.059 | <0.001 | 2.148 | 1.891 to 2.439 | <0.001 |

Abbreviations: BMI, body mass index; CI, confidence interval; CKD-EPI, Chronic Kidney Disease Epidemiology Collaboration; CRP, C-reactive protein; eGFR, estimated glomerular filtration rate; LDL-C, low-density lipoprotein cholesterol; OR, odds ratio; SBP, systolic blood pressure; SGHS, selective glomerular hypofiltration syndrome.

Regression coefficients (β) represent the change in the eGFR_cys_/eGFR_cr_-ratio per unit increase in the exposure. Odds ratios represent the odds of SGHS (eGFR_cys_/eGFR_cr_-ratio <0.7).. Age was modelled per 10 units, BMI per 1 kg/m², smoking as log-transformed pack-years, fasting glucose per 1 mmol/L, SBP per 1 mmHg, and LDL-C per mmol/L. Antihypertensive and lipid-lowering treatments were modelled as binary variables. Study site was included as a fixed effect in all models.

| **Variable** | **Included (n=28,078)** | **Excluded due to missing covariates (n=1,168)** | **Participants with missing values for creatinine or cystatin C (n=908)** |
| --- | --- | --- | --- |
| **Age, years - mean (SD)** | 57.5 (4.3) | 57.4 (4.3) | 57.6 (4.3) |
| **Male Sex - % (n)** | 48.7 (13684) | 54.5 (636) | 34.0 (309) |
| **BMI, kg/m^2^ – median (Q1-Q3)** | 26.3 (23.9–29.4) | 27.0 (24.5–30.3) | 26.6 (23.9-30.1) |
| **Systolic blood pressure, mmHg - median (Q1-Q3)** | 124 (114–136) | 127 (116–140) | 123 (112-136) |
| **HbA1c, mmol/mol - median (Q1-Q3)** | 36.0 (33–38) | 36.0 (34–39) | 36.0 (34-39) |
| **Fasting glucose, mmol/L - median (Q1-Q3)** | 5.5 (5.0–5.9) | 5.5 (5.1–6.1) | 5.4 (5.0-5.9) |
| **eGFR_cys_/eGFR_cr_-ratio (CKD-EPI) - median (Q1-Q3)** | 0.92 (0.80–1.05) | 0.91 (0.79–1.03) | ---- |

Supplementary table 15. Characteristics of excluded participants in comparison to included participants

Abbreviations: BMI, body mass index; eGFR, estimated glomerular filtration rate; SBP, systolic blood pressure; The excluded group comprised participants with missing data in at least one covariate. Therefore, the number of observations may vary slightly between variables
